# Effect of Polyethylene Glycol Additive on the Structure and Performance of Fabric-Reinforced Thin Film Composite

**DOI:** 10.3390/molecules28052318

**Published:** 2023-03-02

**Authors:** Xiao Wang, Yuntao Zhao, Xueyou Wen

**Affiliations:** 1 Chongqing Institute of Green and Intelligent Technology, Chinese Academy of Sciences, Chongqing 400714, China; 2 School of Water Resources and Environment, Hebei GEO University, Shijiazhuang 050031, China; 3 Hebei Key Laboratory of Sustained Utilization and Development of Water Resources, Shijiazhuang 050031, China

**Keywords:** thin film composite, polyethylene glycol, forward osmosis, polysulfone, fabric

## Abstract

Fabric-reinforced thin film composite (TFC) membranes exhibit outstanding mechanical durability over free-standing membranes for commercial applications. In this study, polyethylene glycol (PEG) was incorporated to modify the polysulfone (PSU) supported fabric-reinforced TFC membrane for forward osmosis (FO). The effects of PEG content and molecular weight on the structure, material property and FO performance of the membrane were investigated comprehensively, and the corresponding mechanisms were revealed. The membrane prepared by using 400 g/mol PEG exhibited better FO performances than those of membranes with 1000 and 2000 g/mol PEG, and 20 wt.% was demonstrated to be the optimal PEG content in the casting solution. The permselectivity of the membrane was further improved by reducing the PSU concentration. The optimal TFC-FO membrane had a water flux $\left(J_{w}\right)$ of 25.0 LMH using deionized (DI) water feed and 1 M NaCl draw solution, and the specific reverse salt flux ($J_{s}$/$J_{w}$) was as low as 0.12 g/L. The degree of internal concentration polarization (ICP) was significantly mitigated. The membrane behaved superior to the commercially available fabric-reinforced membranes. This work provides a simple and low-cost approach in the development TFC-FO membrane and shows great potential in the large-scale production for practical applications.

## 1. Introduction

FO has been widely regarded as a valuable alternative process for water treatment. It holds several advantages, including mild operating conditions, low requirements for pretreatment, high water recovery, efficient contaminant rejection, low membrane fouling propensity and good fouling reversibility, over the conventional thermally-driven and pressure-driven membrane separation technologies, especially in the applications for seawater desalination, high value-added product concentration and treatment of complex and impaired wastewater, high salinity and high fouling potential streams [1,2,3,4,5,6].

As the core of FO process, the lack of high-performance FO membrane is the major bottleneck restricting the development of FO technology [7]. An ideal FO membrane material should achieve high water permeability, high solutes selectivity and be mechanically robust. Current developed FO membranes mainly include integrally asymmetric, layer-by-layer assembled and TFC membranes [8,9,10]. Among them, TFC is the most attractive FO membrane, owing to its high permselectivity, acid-base resistance and biological stability [11]. The two-layer structured TFC membrane is typically composed of an active layer (i.e., separation layer) and a porous support layer [12,13]. The ultra-thin dense active layer that is mainly prepared by interfacial polymerization contributes the permselectivity of the TFC membrane, while the underneath porous layer acts as an essential mechanical support and provides a physical interface for the formation of the active layer. It has been reported that the ICP in the support layer is the main factor in resisting water permeation across the FO membrane [7,14]. The degree of ICP is determined by the thickness, hydrophilicity, porosity, pore structure and penetrability of the substrate, which can be quantitatively described by structural parameter (*S*). Several modification strategies on the support layer and active layer have been proposed to reduce the ICP and improve FO performances of the membrane. The main methods for support layer optimization include: (1) utilization of novel materials with excellent mass transfer performance, such as a nanofiber membrane [15], vertically oriented porous substrate [16] as porous support layer; and (2) improvement in the structure, physical and chemical properties of the traditional support layer, such as constructing vertical finger-like macrovoids that span the entire support layer [17], hydrophilicity modification by means of blending or doping [18,19]. In terms of the active layer, strategies mainly include: (1) chemical modification, such as using new types of polyamines or polyacyl chloride monomers [20], surface grafting [21]; and (2) incorporation of nanomaterials, including graphene oxide [22], carbon nanotubes [23], graphene quantum dots [24] and water channel proteins [25]. The degree of ICP is significantly reduced, and the permeability of the membranes is greatly improved. However, these FO membranes are prepared using free-standing polymeric substrates without any fabric support. They exhibit remarkably low mechanical strength and are not practical for commercial applications, especially for the large-scale production of FO membranes and components [26]. The complex preparation process of these TFC-FO membranes with low production efficiency also limits their practical uses. Therefore, it is critically important to develop high osmosis performance and mechanically robust membranes. Currently, combining the polymeric functional layer with tough fabric is one of the most effective strategies, although the permselectivity could be compromised [27,28]. Among the functional polymers, PSU is a commonly commercially affordable membrane material with good physical and chemical stability and biological stability. It is currently one of the most important polymeric materials as the porous support layer of the TFC membrane. Recently, Ren et al. [18] prepared polyester (PET) nonwoven fabric reinforced PSU/postsulfonated PSU substrate; the use of PET in the FO membrane brought in good mechanical properties. However, the nonwoven fabric could cause severe ICP in the membrane [18,28]. Nguyen et al. [11] indicated that woven fabric is more suitable to reinforce the FO membrane substrate compared with nonwoven fabric. Han et al. [29] firstly integrated sulfonated material with woven fabric to develop high performance fabric-reinforced TFC-FO membranes. In our previous work, PET woven fabric was embedded in PSU/sulfonated PSU (sPSU) substrate [26]. The TFC membrane exhibited excellent FO performance with low structural parameter, indicating that the degree of ICP was effectively mitigated. The tensile strength and Young’s modulus were significantly larger than those of fabric-free membranes, imparting impressive mechanical durability. Nevertheless, the preparation of the membranes involved complex copolymerized sulfonation and polymer blending, which was uneconomic. Up to today, there are very limited reports on fabric-reinforced TFC-FO membranes. A simple and low-cost approach is needed in the development of commercially available membranes.

PEG is a commonly used additive for the casting solution system in the preparation of phase inversion membranes. It could change the morphology and structure of the prepared membrane by altering the thermodynamic and rheological properties and gel dynamic behavior of the casting solution. However, the addition of PEG in the fabric-reinforced TFC-FO membrane has not been reported yet. In this study, PEG was used to modify the fabric-reinforced PSU substrate. The effects of PEG content and molecular weight as well as PSU concentration on the structure and osmosis performance of the TFC-FO membrane were systematically investigated, and the corresponding mechanisms were revealed. Since PEG could diffuse and dissolve into the coagulation bath during phase inversion, the composition of the formed support layer only contained PSU. We propose an easy-to-process way of manufacturing PSU/PEG supported FO membranes from abundant chemicals, which is economically beneficial. Moreover, the membrane materials mainly contain low-cost PSU and PEG without incorporating expensive substances such as nanomaterials, water channel proteins that are used in the other strategies. Therefore, this paper provides an economic strategy with easy processability for the development of high-performance fabric-reinforced TFC-FO membranes.

## 2. Results and Discussion

**Figure 1:**
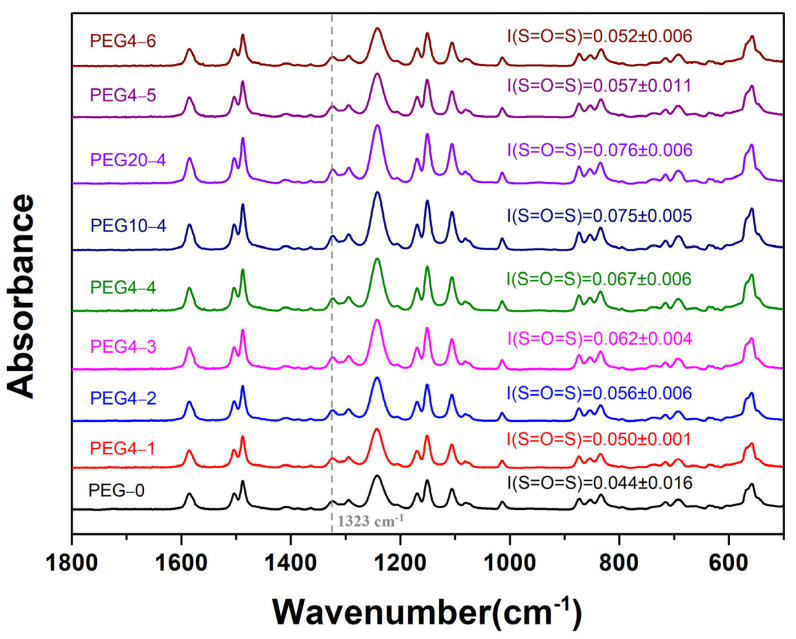
FTIR spectra of PSU/PEG substrate surface.

The intrinsic property of the TFC membrane substrate is one of the key factors that affect its ICP and the formation and performance of the active layer. In this paper, a series of woven fabric-reinforced PSU/PEG membrane substrates were prepared according to the casting solution composition in Table 1, and their physical and chemical properties including porosity, mean surface pore size, surface contact angle, thickness and surface chemical composition, were characterized as shown in Table 2 and Figure 1. The substrate consisted of a porous layer and a fabric layer. Our previous research showed that in the preparation of the additive-free woven fabric-reinforced PSU substrates, high-quality and low-defect coatings on the woven fabrics were achieved when the PSU concentration in the casting solution was 15 wt.% [26]. As a non-solvent additive, the incorporation of PEG increases the viscosity of the casting solution when the PSU concentration is constant [30]. In this study, the casting solution viscosity increased with the increase in PEG content, and the resultant substrates showed good quality when the PEG concentration was up to 20 wt.%. However, excess PEG incorporation impaired the quality of the substrate due to the increased high viscosity that was not conducive to the mixing and spreading of the casting solution. Furthermore, casting solution with PSU concentration of 12 wt.% and 9 wt.% was prepared when PEG content maintained at 20 wt.%. The substrates still exhibited good quality as the viscosity of the casting solution was appropriate. However, the quality significantly decreased when the PSU concentration was below 9 wt.%, where the solution viscosity was too low for the substrate formation.

The addition of PEG increased the porosity of the PSU substrate, compared with the control substrate (PEG-0), as shown in Table 2. It has been reported that the PEG in the casting solution facilitated the pore formation of the substrate during the NIPS [31,32]. However, the porosity was almost unchanged when the PEG content exceeded 10 wt.%. The increase in PEG content increased the viscosity of the casting solution, which slowed down the rate of the outward diffusion of the solvent and the inward diffusion of the non-solvent in the coagulation bath [33,34]. Thus, the NIPS rate of the casting solution was reduced, which was not conducive to the increase in the membrane porosity. Moreover, the porosity of the porous layer of PEG4-4, PEG10-4 and PEG20-4 was 84.6, 84.7 and 84.6, respectively, which indicated that the molecular weight of PEG did not affect the porosity under a certain PSU concentration. By reducing the PSU content, the porosity was increased as observed in PEG4-4 (PSU: 15 wt.%), PEG4-5 (PSU: 12 wt.%) and PEG4-6 (PSU: 9 wt.%), whose porosity was 84.6, 85.7 and 89.5, respectively. Compared with the porous layer, both the warp and weft yarns of the woven fabric layer did not have porous structures. Therefore, the porosity of the overall substrate was lower than that of its porous layer. The porosity of the fabric-reinforced substrate was affected by both the porosity of its porous layer and the volume proportion of its woven fabric.

The surface pore size of the substrate is critical in the formation of the active layer of the TFC-FO membrane. As shown in Table 2, the average surface pore diameter, *d_p_*, of the substrate was between 7–24 nm, which was within the pore size range of the ultrafiltration membrane. It was conducive to the formation of the high-quality active layer. The *d_p_* of PEG-0 was 19.0 nm. Under a constant PSU concentration, the *d_p_* decreased with the increase in PEG content and molecular weight in the casting solution. It resulted from the increased viscosity of the casting solution at higher PEG content or molecular weight. As discussed before, the increase in the viscosity slowed down the NIPS rate. It facilitated the formation of dense and small sized pores. However, at constant PEG content and molecular weight, the *d_p_* increased with the decrease in PSU concentration, which could be related to the reduced viscosity at low PSU concentration.

The surface hydrophilicity/hydrophobicity of the substrate is another important factor affecting the formation of the active layer of the TFC-FO membrane, and it can be investigated by means of water contact measurement. For porous materials, it should be pointed out that the measured contact angle is an apparent value, depending on pore size, pore size distribution, roughness and chemical nature of the substrate. It can be seen from Table 2 that for the same substrate, its surface contact angle in the wet state was smaller than that in the dry state. In the dry state, the surface contact angle of the prepared PSU/PEG substrate was similar to that of the PEG-free substrate. In contrast, in the wet state, the contact angle of the PSU/PEG substrate is slightly lower than that of the PEG-0. This could result from the effect of the PEG on the surface roughness of the substrate. In general, the content and molecular weight of PEG did not significantly influence the contact angle of the substrate, because most of the PEG would dissolve into the conjugation bath during the phase conversion course. The hydrophilicity of the prepared PSU/PEG substrates could be comparable.

The ICP of a TFC-FO membrane is positively related to its substrate thickness. Table 2 shows that the overall thickness of the prepared PSU/PEG membranes was 59–67 μm, including the thickness of the woven fabric (about 50 μm). The thickness of the porous layer was only 9–17 μm, approximately. Due to the use of double-blade co-casting method, the ultra-thin and low-defect coating was prepared on the surface of the woven fabric. The thickness of the porous layer was effectively controlled. It should be noted that there was a difference in the thickness of the prepared substrates, especially when PEG20 was incorporated. Under a certain PSU concentration, the thickness of the PSU/PEG substrate increased with the increase in PEG molecular weight. The coating amount of the polymer per unit area tended to be increased with the increase in the viscosity of casting solution under the same casting knife thickness and speed, hence increasing the substrate thickness.

The porous support layer of the substrates exhibits a typical asymmetric structure, which is composed of a dense skin layer, a spongy-like microporous structure and a finger-like macroporous structure. ATR-FTIR was used to analyze the surface chemical composition of the substrates, and the FTIR spectra are shown in Figure 1. A characteristic absorption peak, corresponding to the sulfone group (S=O=S), was observed at ~1323 cm^−1^ in each FTIR spectrum. The peak intensity of the sulfone group was 0.044 in the PEG-0 substrate. At a certain PSU concentration, the intensity increased with the increase in PEG content (PEG-0, PEG4-1, PEG4-2, PEG4-3, PEG4-4) or molecular weight (PEG4-4, PEG10-4, PEG20-4). According to the positive correlation between the peak intensity of the sulfone group and its content, the increase in the PEG content or molecular weight could result in a denser surface structure and increase the thickness of the skin layer and the spongy-like microporous structure. Thus, the PSU content at the substrate surface was increased. Furthermore, under a certain PEG4 content at 20 wt.%, the peak intensity decreased with the decrease in the PSU concentration (PEG4-4, PEG4-5, PEG4-6), which may be relative to the reduced sulfone content and thickness of the surface layer.

**Figure 2:**
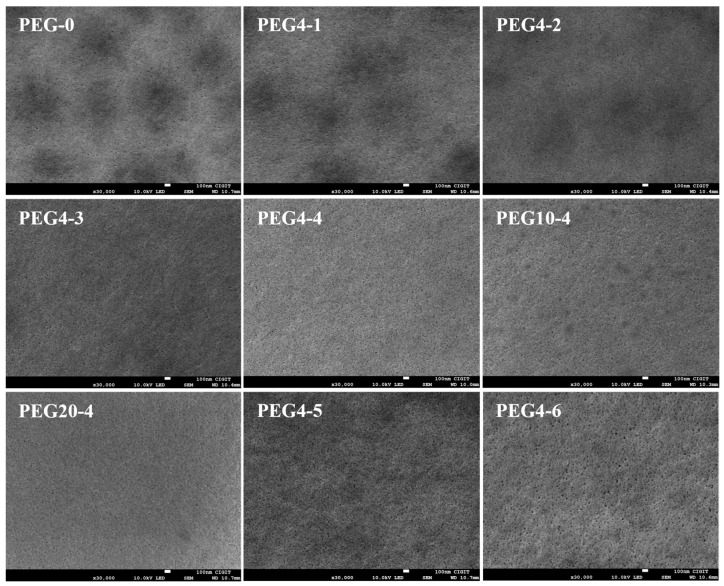
SEM images for the top surfaces of PSU and PSU/PEG substrates.

**Figure 3:**
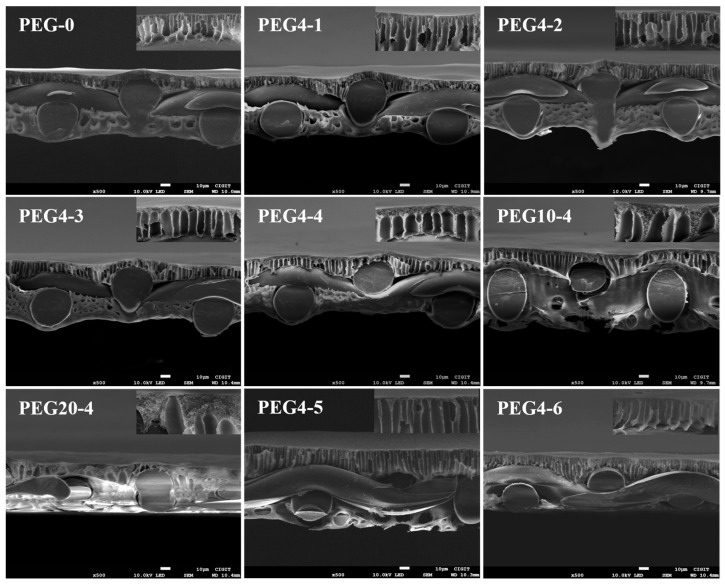
SEM images for the cross-section of PSU and PSU/PEG substrates. The insets reveal the morphologies of the substrates near the top surface at higher magnification.

**Figure 4:**
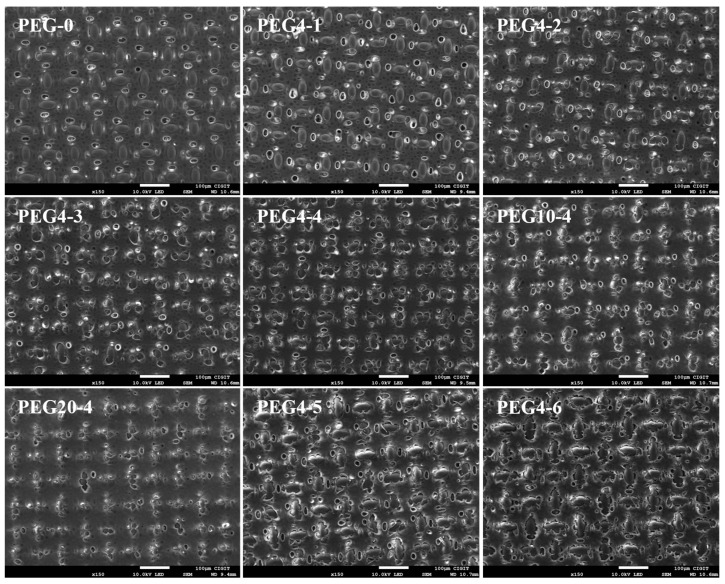
SEM images for the bottom of PSU and PSU/PEG substrates.

The surface, cross-section and bottom morphology of the prepared PSU/PEG substrates are shown in Figure 2, Figure 3 and Figure 4, respectively. The surface morphology (Figure 2) of the prepared PSU and PSU/PEG substrates was similar, and the surface pore diameter was at the nanometer level, which was consistent with the pore size analysis results. However, a few concavities (darker area) in microscale appeared on the surface of the PEG-0 substrate. In the dry state for SEM observation, the surface layer of the substrate tended to shrink and collapse to some extent, which resulted in the formation of the concavities. The thinner the surface layer was, the greater degree of the collapse was. With the increase in the PEG content, the number of the concavities gradually decreased and disappeared when the PEG content was over 10 wt.%. This could result from the increased thickness of the surface layer, including the skin layer and the spongy-like microporous structure, at higher PEG incorporation.

It can be observed in Figure 3 that the woven fabrics were embedded in the porous support layers instead of being cushioned at their bottom for the PSU/PEG substrates. Some of the finger-like macropores were cut off by the warp and weft yarns of the woven fabrics, and only the macropores located in the openings of fabrics penetrated through to the bottom of the substrate, which may increase the pore curvature of the substrate. It is worth noting that the cross-section morphology of the porous support layer under different PEG content or molecular weight was different. It can be clearly observed from the insets in Figure 3 that at the same PSU concentration, the thickness of the spongy-like micropore structure increased with the increase in the PEG content or molecular weight, while the number of the finger-like macropore structure decreased, and the macropore channel became wider. For the substrates with 20 wt.% PEG4, the thickness decreased with the decrease in the PSU content. They were consistent with the FTIR results. Compared with the PEG-0 substrate, the porous support layer in the PSU/PEG substrate exhibited better macropore channel connectivity with fewer closed capsule-like macropores. The macropore channels were uniform for most of the PSU/PEG substrates. According to the NIPS mechanism, the cross-section morphology of the porous support layer is determined by the thermodynamic and kinetic properties during the NIPS course. When the primary film was immersed in the coagulation bath, the casting solution solvent and coagulation agent inter-diffused. With the increasing amount of coagulation agent into the casting solution, liquid–liquid phase separation occurred in the casting solution, forming a polymer-poor phase and a polymer-rich phase. Delayed liquid–liquid phase separation was often conducive to the formation of a sponge-like micropore structure, while instantaneous liquid–liquid phase separation was often conducive to the formation of a large macropore structure [17,35,36]. After the liquid–liquid phase separation of the casting solution, the mutual diffusion between the casting solution solvent and the coagulation agent continued. The polymer-rich phase was solidified to form a film during crystallization, gelation or vitrification. When the growth rate of the polymer-poor phase was lower than the solidification rate of the polymer-rich phase, the formation of the macroporous structure was inhibited, otherwise it was conducive to the macro pore formation [17,34]. Due to the poor compatibility between PSU and PEG, the addition of PEG in the casting solution reduced the thermodynamic stability of the casting solution, and the solubility of PEG in NMP decreased with the increase in its molecular weight [34]. Therefore, when the PSU concentration was constant, the thermodynamic stability of PSU/PEG/NMP casting solution decreased with the increase in PEG content or molecular weight, which was conducive to the liquid–liquid phase separation. The growth rate of the polymer-rich phase was slower than the solidification rate of the polymer-rich phase. However, the viscosity of the casting solution increased with the increase in PEG content or molecular weight. The increased viscosity could slow down the rate of the outward diffusion of the casting solution solvent and the inward diffusion of the coagulation agent, which was unfavorable to the liquid–liquid phase separation and reduced the growth rate of the polymer-poor phase.

Figure 4 shows that there were numerous macro size pores on the bottom surface of each substrate, and the presence of these pores could facilitate the solution to enter and exit the substrate. By means of statistical analysis, it was found that the number of the macropores increased with the increase in the PEG content but decreased with the increase in its molecular weight. PEG4 substrates may exhibit better permeability than PEG10 and PEG20 substrates. Moreover, at a certain PEG4 content of 20 wt.%, the number of the pores first increased and then decreased with the decrease in the PSU concentration. PEG4-5 possessed the largest number of pores, compared with PEG4-4 and PEG4-6. It can be seen from Figure 4 that most of the woven fabrics, excluding the intersections of warp and weft yarns, were embedded in the porous support layer, which ensured the firm combination of the woven fabric and the porous support layer.

**Figure 5:**
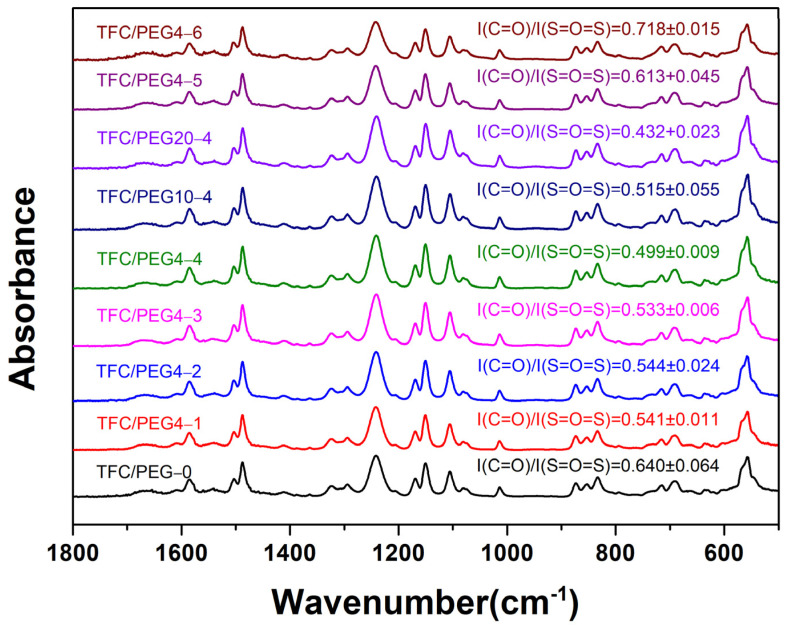
FTIR spectra of PSU/PEG supported TFC-FO membranes.

The surface chemical composition of the prepared TFC-FO membranes was analyzed by ATR-FTIR, and the results are shown in Figure 5. Two characteristic peaks at ~1663 and ~1541 cm^−1^, corresponding to the amide I (C=O stretching vibration) and amide II (C-N stretching and in-plane N-H bending vibration), respectively, were observed. This indicated that PA active layers were successfully synthesized on the surface of the prepared PSU/PEG substrates through interfacial polymerization. Since the detection depth of ATR-FTIR measurement was greater than the thickness of the PA active layer, the characteristic peak at ~1323 cm^−1^ for the sulfone groups (S=O=S) in the substrate also appeared. The thickness of the PA active layer could be analyzed by using the characteristic absorption peak intensity ratio of amide and sulfone groups in the FTIR spectrum of each membrane. In general, a higher ratio of I (C=O)/I (S=O=S) indicates a thicker PA layer [11,37]. It can be seen from Figure 5 that the intensity ratio for TFC/PEG-0 was 0.64, which was higher than that for the TFC/PEG membranes with 15 wt.% PSU. It implied that the incorporation of PEG could reduce the thickness of the PA layer. A thinner PA layer could result in lower resistance to salt and water transport. However, there was no big difference of the ratio for the membranes with varied PEG content or molecular weight. The PEG content and molecular weight may not significantly affect the formation and the thickness of the active layer. In contrast, for the membranes with 12 and 9 wt.% PSU, the intensity ratio increased dramatically. In this case, the analysis of PA layer thickness by intensity ratio could be invalid, because the intensity of sulfone groups reduced notably with the decrease in PSU concentration.

**Figure 6:**
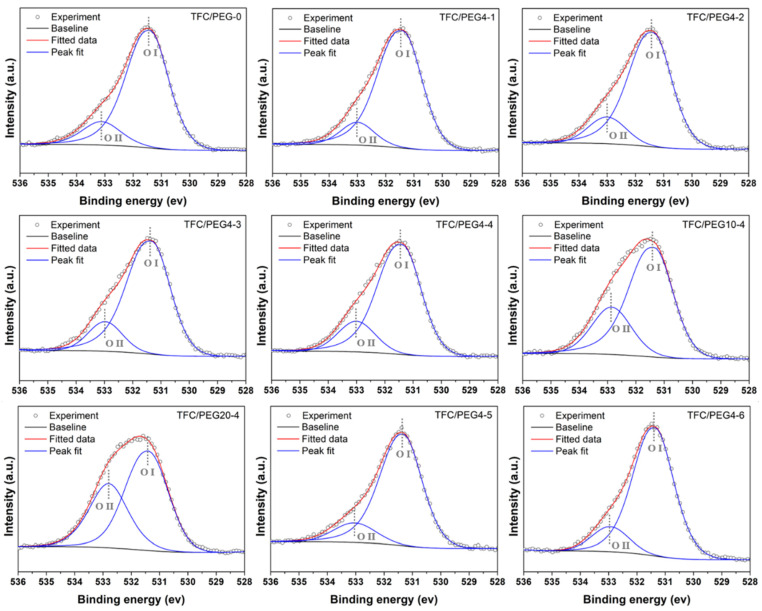
Convoluted high-resolution O 1s spectra of PSU/PEG supported TFC-FO membranes.

The PA active layer of the TFC membrane was synthesized by MPD and TMC. It was composed of a linear portion that contained free pendant -COOH hydrolyzed from the unreacted -COCl and a cross-linked portion where -COCl was all involved in a cross-linking reaction with -NH_2_. The cross-linking degree was important in determining the water flux and salt rejection of TFC membranes [11,38]. Since the detection depth of XPS was less than the thickness of the active layer, XPS was used to investigate the surface element composition, content and cross-linking degree of the active layer. The results are shown in Figure 6 and Table 3. It can be seen from Table 3 that the active layer was mainly composed of carbon, oxygen and nitrogen, which was consistent with the element composition of PA. The oxygen atoms were from -CONH- (N-C=O: O I, ~531.6 ev) and -COOH (O-C=O: O II, ~533.0 ev), while nitrogen atoms were from -CONH- and -NH_2_. A higher O I/O II content ratio corresponded to a smaller O/N content ratio. Theoretically, the O/N ratios of fully cross-linked and fully linear PA were 1.0 and 2.0 respectively. Thus, the cross-linking degree of the PA layer can be estimated by the O/N ratio and O I/O II ratio. The closer the ratio was to 1.0, the higher the cross-linking degree of the PA layer was. In Table 3, the O/N ratio of TFC/PEG-0 was 1.18, indicating a highly cross-linked active layer. The ratio increased with the increase in the PEG content and its molecular weight. This could mainly result from the pore size of the substrate. As shown in Table 2, the pore diameter of the substrate decreased with the increase in the PEG content and its molecular weight. During the IP course, the smaller pores on the substrate surface absorbed less amount of MPD solution that migrated to reaction interface for the following IP with TMC. Thus, a less cross-linked PA layer was favored with higher O/N ratio. TFC/PEG20-4 exhibited the highest O/N ratio among the membranes. In contrast, with the decrement in PSU content from 15 to 12 wt.%, the pore size of the substrate increased. It facilitated sufficient MPD supply to the reaction zone, resulting in a notable increase in the cross-linking degree. The O/N ratio of TFC/PEG4-5 was as low as 1.13, compared with that of TFC/PEG4-4. However, the ratio of TFC/PEG4-6 was slightly raised with further decline in the PSU content to 9 wt.%. The excess MPD absorbed in its big pores could adversely affect the active layer formation [39].

**Figure 7:**
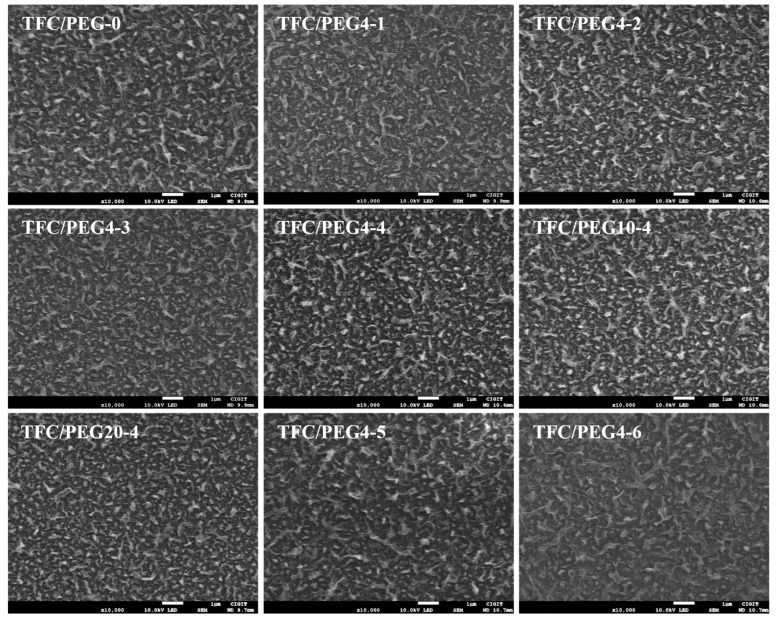
SEM images for top surface of PSU/PEG supported TFC-FO membranes.

**Figure 8:**
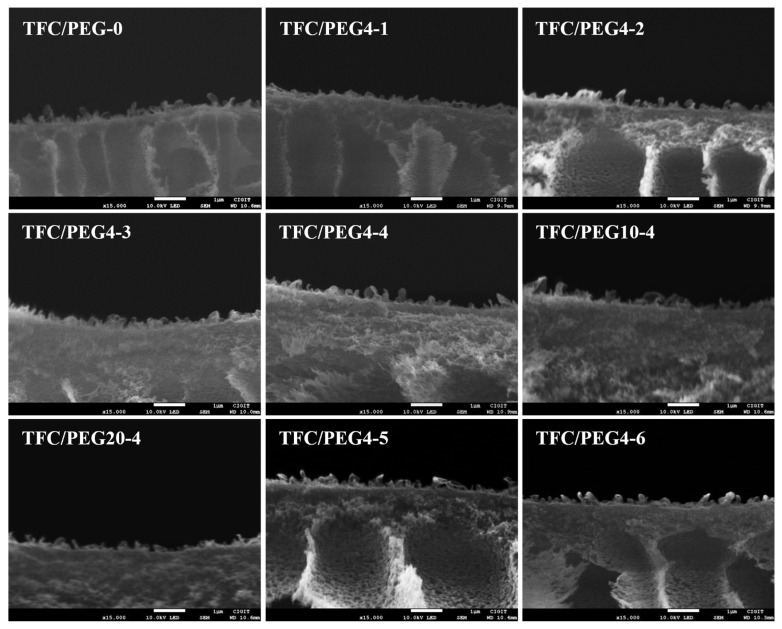
SEM images for cross-section of PSU/PEG supported TFC-FO membranes.

**Figure 9:**
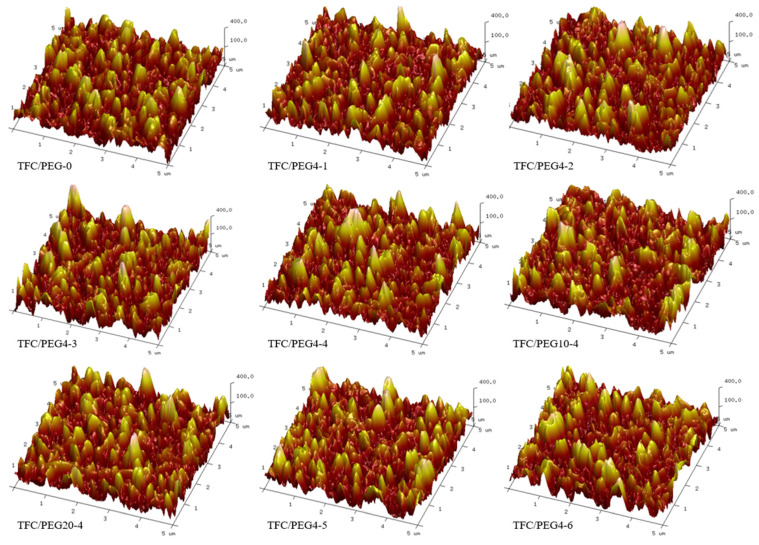
AFM images for PSU/PEG supported TFC-FO membranes.

The surface and cross-section morphologies of the TFC-FO membranes were investigated by SEM as shown in Figure 7 and Figure 8, respectively. The surface of each membrane presented a typical ridge-and-valley structure. There was no significant difference in terms of the surface morphologies of the membranes prepared at different PEG content or molecular weight. The active layer was firmly combined with its porous support layer. The ridge-and-valley structure of the active layer was further confirmed from AFM images as displayed in Figure 9. The corresponding surface roughness (R_q_ and R_a_) of each membrane was obtained and listed in Table 4. In general, the fouling resistance of a TFC membrane is negatively correlated with its surface roughness, while the water permeability coefficient is positively correlated with the roughness. The R_q_ and R_a_ of TFC/PEG-0 was 93.6 and 75.2, respectively. The incorporation of PEG in the casting solution of the substrate did not significantly influence the surface roughness of the active layer under the same interfacial polymerization conditions.

**Figure 10:**
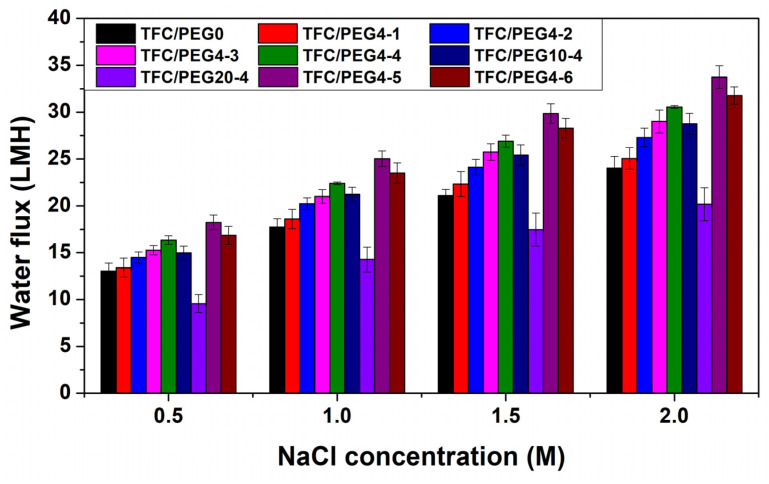
Water flux of PSU/PEG supported TFC-FO membranes.

**Figure 11:**
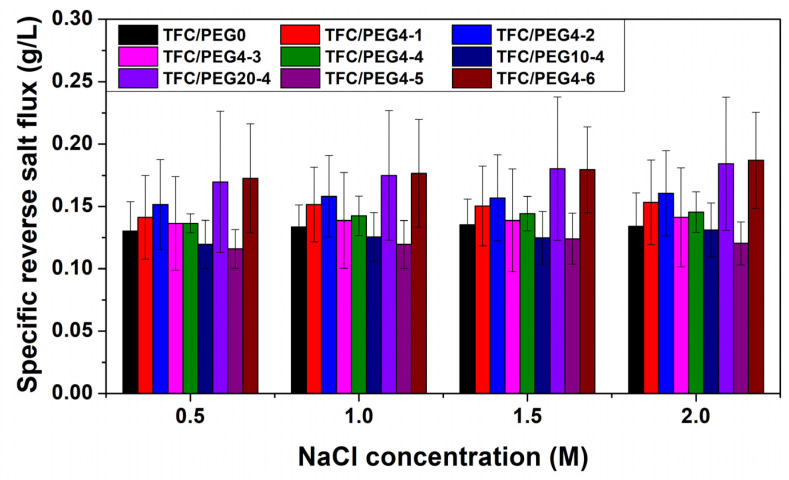
Specific reverse salt flux of PSU/PEG supported TFC-FO membranes.

The FO performances, including $J_{w}$, $J_{s}$ and $J_{s}$/$J_{w}$, were measured at different draw solution concentrations, and the results are presented in Figure 10 and Figure 11 and Figures S1–S4. The transport properties, including *A*, *B*, *A*/*B* and *S*, of the prepared TFC-FO membranes were calculated by fitting the measured $J_{w}$ and $J_{s}$. The values were listed in Table 5 and Table S1. Generally, the *A*/*B* and $J_{s}$/$J_{w}$ can reflect the permselectivity of FO membrane. *A*/*B* could be mainly affected by the active layer thickness and cross-linking degree. Higher *A*/*B* and lower $J_{s}$/$J_{w}$ means better permselectivity of the formed PA layer and less draw solution loss in FO. The *S* value quantifies the ICP extent in the membrane, which is critical for membrane design. The *S* value of TFC/PEG-0 was 363 μm. Incorporation of PEG in the casting solution reduced the structural parameter. It further reduced with the increase in PEG content, which was mainly related to the changes in the properties of the membrane substrate. As discussed in the SEM morphology, under the same PSU concentration, the thickness of spongy-like microporous structure in the substrate increased with the increase in PEG content, while that of the finger-like macroporous structure decreased relatively. Previous studies have indicated that sponge-like micropore structure is more likely to cause ICP than finger-like macropore structure [17,27]. Additionally, the curvature of sponge pore is normally greater than that of finger pore. Nevertheless, we believe that the curvature of sponge pore could be reduced in the case of high interpore connectivity. Feng et al. [40] demonstrated that the addition of PEG in the casting solution could increase the interpore connectivity in the sponge structure of the substrate that was formed during NIPS. It contributed to the decrease in structural parameter.

In can be observed in Figure 10 that the water flux of all TFC-FO membranes increased with the concentration of the draw solution, because the driving force for water transfer in the FO process was positively correlated with the draw solution concentration. In contrast, the specific reverse salt flux of the membranes was hardly affected by the draw solution concentration as shown in Figure 11. The prepared FO membranes exhibited good integrity. Under the same operation condition, the water flux of the TFC-FO membrane decreased with the increase in PEG molecular weight in the casting solution, which may be mainly related to the molecular composition of the active layer. The PA layer may contain ester groups, especially when the PEG with high molecular weight was used in the casting solution. The increase in the number of hydrophobic ester groups in the active layer was not conducive to the solution-diffusion behavior of water molecules, resulting in the decrease in the water permeability coefficient. Thus, the *A* value of the membranes decreased with the increase in PEG molecular weight in the casting solution. Figure 10 and Figure S1 shows that when the molecular weight of PEG was 400 and 1000 g/mol, the water flux of the membranes increased with the increase in PEG content, which was due to the improvement of their water permeability coefficient and structural parameter. The $J_{w}$ of TFC/PEG4-4, containing 20 wt.% PEG in casting solution, was 22.4 LMH when using 1 M NaCl as draw solution. However, when the molecular weight of PEG was 2000 g/mol, the water flux of the membranes first increased and then decreased with the increase in PEG content. All the PEG20 membranes showed lower water flux compared with that of TFC/PEG-0. Although their *S* value was lower than that of the control, their *A* value was quite low. As indicated in Table 3, the O/N ratio of PEG20-4 was significantly higher than that of TFC/PEG-0, indicating a low cross-linking degree. The insufficient cross-linked PA layer could result in a low osmotic pressure and thus a low water permeation under FO operation. In terms of specific reverse salt flux, overall, the $J_{s}$/$J_{w}$ value of TFC/PEG4 was slightly lower than that of TFC/PEG10 and TFC/PEG20 under the same operation conditions. This could result from the influences of both PA layer thickness and cross-linking degree.

Overall, TFC/PEG4 exhibited better FO performances over TFC/PEG10 and TFC/PEG20. A 20 wt.% was demonstrated to be the optimal PEG content in the casting solution. Moreover, the water flux of the membrane was improved by reducing the PSU concentration from 15 wt.% to 12 wt.%. The $J_{w}$ of TFC/PEG4-5 was 25.0 LMH using 1 M NaCl as draw solution, and the $J_{s}$/$J_{w}$ was as low as 0.12 g/L. However, further decrease in PSU concentration (9 wt.%) reduced the $J_{w}$. This could mainly result from the improvement in the substrate structure. As observed in the bottom surface of the substrates in Figure 4, TFC/PEG4-5 possessed a larger number of macro pores that penetrated throughout the substrate, compared with that of TFC/PEG4-4 and TFC/PEG4-6. The pores played a key role in the water permeation. The $J_{w}$ and $J_{s}$/$J_{w}$ of TFC/PEG4-5 in this work is better than that of the reported fabric-reinforced TFC FO membranes [29,41,42,43]. The *S* value of TFC/PEG4-5 and TFC/PEG4-6 membranes were 248.8 and 242.5 μm, respectively (Table 5). The decrease in PSU concentration in the casting solution was beneficial to reducing the ICP of the FO membrane. The *S* value is lower than those of previous reported fabric-reinforced TFC FO membranes [18,29,41,42,43].

## 3. Materials and Methods

### 3.1. Materials

PEG4 (400 g/mol, chemically pure), PEG10 (1000 g/mol, chemically pure), PEG20 (2000 g/mol, chemically pure) and sodium chloride (NaCl, analytical reagent) were purchased from Sinopharm Chemical Reagent Co., Ltd. PSU (Duel P-3500, 77,000–83,000 g/mol) was purchased from Solvay (Shanghai) Co., Ltd. m-phenylenediamine (MPD, analytically pure), trimesoyl chloride (TMC, analytically pure), N-methyl-2-pyrrolidone (NMP, analytically pure) and n-hexane (analytically pure) were provided by Sigma Aldrich (Shanghai) Trading Co., Ltd. PET woven fabric was purchased from Shanghai Bolting Cloth Manufacturing Co., Ltd. DI water (18.25 M Ω·cm) was self-made in the laboratory.

### 3.2. Preparation of TFC Membrane

**Figure 12:**
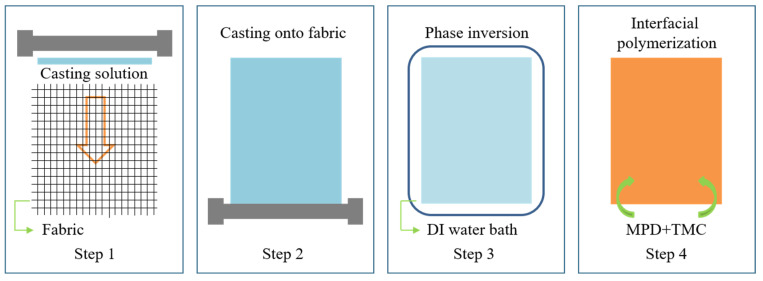
The schematic of the preparation process for PSU/PEG supported TFC-FO membrane.

The fabric reinforced PSU/PEG substrate was fabricated by means of non-solvent induced phase separation (NIPS). PSU and PEG were stirred in NMP solvent at room temperature until it was completely dissolved. The mixture was then placed in a desiccator for degassing for about 24 h, in order to obtain homogeneous and transparent casting solution. The PET woven fabric was flatted and taped on a clean glass plate. The casting solution was uniformly spread on the fabric by a 45 μm casting knife. Then, the primary film was immediately immersed into a 20 °C coagulation bath (DI water) for phase transformation for 10 min. The prepared membrane substrate was rinsed by DI water to completely remove the solvent. The polyamide (PA) active layer was synthesized on the substrate by interfacial polymerization (IP): The substrate was immersed by 2.0 wt.% MPD aqueous solution and soaked for 2 min. After removing the excess MPD solution, 0.1 wt.% TMC/n-hexane solution was placed on the substrate surface for 1 min IP reaction, followed by hexane rinsing to remove the unreacted TMC. The resultant TFC membrane was cured at 60 °C for 3 min, and then stored in DI water at 4 °C for further tests. A schematic of the preparation process of the TFC membrane was depicted in Figure 12, and the images recording the process were presented in Figure S5. The prepared TFC-FO membranes were denoted as TFC/PEG-0, TFC/PEG4, TFC/PEG10 and TFC/PEG20, corresponding to the membrane substrates PEG-0, PEG4, PEG10 and PEG20, respectively.

### 3.3. Characterization

The porosity of the substrate was obtained by a wet–dry weight method. The preserved substrate in DI water was weighed (*m_1_*) after absorbing the excess water on the surface by filter paper. The substrate was then dried under vacuum at 60 °C for 12h and weighed (*m_2_*). Then the substrate was dissolved in NMP to remove PSU. The remaining fabric was cleaned with DI water and further dried under vacuum at 60 °C for 12 h. The weight (*m_3_*) was measured. The porosity, ε(%), was calculated:

(1) $$\varepsilon =\frac{\left(m_{1}-m_{2}\right)/\rho_{w}}{\left(m_{1}-m_{2}\right)/\rho_{w}+\left(m_{2}-m_{3}\right)/\rho_{p}+m_{3}/\rho_{f}}\;$$

where $\rho_{w}$, $\rho_{p}$ and $\rho_{f}$ represent the density of water, PSU and fabric, respectively. A droplet shape analyzer (DSA100, KRUSS, Germany) was used to measure the water contact angles on the surface of dry and wet substrate. The droplet volume was 3 μL, and the contact angles were measured by sessile drop method. The thickness of the substrate was measured with a bench thickness meter (CH-12.7-STSX, Shanghai Liuling Instrument Factory, Shanghai, China) in wet condition. The mean pore diameter, $d_{p}$, of the substrate was measured by a liquid–liquid displacement method (PRM-1200GL, Benelux Scientific, Ede, Netherlands). The surface tension of the wetting solution was 1.7 mN/m. Atomic force microscopy (AFM, Dimension Icon, Bruker, Santa Barbara, CA, USA) was used to characterize the surface morphology and roughness of the TFC-FO membranes. The tap mode was selected, and the scan range was 5.0 μm × 5.0 μm. Scanning electron microscopy (SEM, JSM-7800F, JEOL) was used to characterize the surface, cross-section and bottom morphology of the substrates and TFC-FO membranes. The samples for cross-sectional view were placed in liquid nitrogen and carefully cut by a sharp razor knife. All the samples were fully dried and coated by gold before the SEM observation. Attenuated total reflection Fourier transform infrared spectroscopy (ATR-FTIR, Cary630, Agilent) was used to characterize the surface chemistry of the substrates and TFC-FO membranes with resolution of 2 cm^−1^. X-ray photoelectron spectroscopy (XPS, Esca Lab 250Xi, Thermo Fisher Scientific, Waltham, MA, USA) was used to analyze the elements on the surface of the TFC-FO membranes.

The water flux ($J_{w}$), reverse salt flux ($J_{s}$) and specific reverse salt flux ($J_{s}$/$J_{w}$) of the prepared TFC-FO membrane was obtained by a custom cross-flow FO system. The effective test membrane area of the FO unit was 42 cm^2^. During the test, the FO mode (active layer against feed solution) was selected, where the cross-flow velocity of the feed solution (DI water) and the draw solution (NaCl solution) was 6.4 cm/s. The concentration of the NaCl draw solution included 0.5 mol/L, 1.0 mol/L, 1.5 mol/L and 2.0 mol/L. All the tests were conducted at 25 ± 1 °C for 30 min, and the changes of the conductivity of the feed solution and the weight of the draw solution were recorded every 2 min by a conductivity meter (DDSJ-308A, INESA Scientific Instrument, Shanghai, China) and balance (BSA6202S-CW, Sartorius), respectively. The $J_{w}$ (Lm^−2^h^−1^ or LMH) was calculated according to Equation (2):

(2) $$J_{w}=\frac{∆V}{∆tA_{m}}\;$$

where ∆*V* (L) is the volume of permeate water during ∆*t* (h), and *A_m_* (m^2^) is the effective membrane test area. The *J_s_* (gm^−2^ h^−1^ or gMH) was calculated via Equation (3):

(3) $$J_{s}=\frac{C_{t}V_{t}}{A_{m}∆t}$$

where *C_t_* (g/L) and *V_t_* (L) are the concentration of the NaCl draw solute and the volume of the feed solution over ∆*t*, respectively. The *C_t_* was obtained according to a standard draw solution concentration–conductivity curve. According to the method reported by Tiraferri et al. [41], the water permeability coefficient (*A*), salt permeability coefficient (*B*) and structural parameter (*S*) of the prepared TFC-FO membrane were calculated by fitting the measured $J_{w}$ and $J_{s}$ at four draw solution concentrations.

## 4. Conclusions

In this work, a series of PEG modified fabric-reinforced PSU TFC-FO membranes were prepared. The incorporation of the non-solvent additive PEG could change the thermodynamic and gel kinetic properties of PSU/NMP casting solution, thus affecting the porosity, pore structure, hydrophilicity, thickness, cross-linking degree and roughness of the membrane. The influences of the PEG content and molecular weight as well as PSU concentration on the FO performances and transport properties were systematically investigated. Appropriate PEG addition effectively decreased ICP and optimized the permselectivity of the membrane. $J_{w}$ of TFC/PEG4-5 was 25.0 LMH using 1 M NaCl as draw solution, and the $J_{s}$/$J_{w}$ was as low as 0.12 g/L.

## Figures and Tables

**Table 1:** Composition of PSU/PEG/NMP casting solutions.

Substrate Code	Composition (wt.%)		
	PSU	PEG	NMP
PEG-0	15	0	85
PEG4-1, PEG10-1, PEG20-1	15	5	80
PEG4-2, PEG10-2, PEG20-2	15	10	75
PEG4-3, PEG10-3, PEG20-3	15	15	70
PEG4-4, PEG10-4, PEG20-4	15	20	65
PEG4-5	12	20	68
PEG4-6	9	20	71

**Table 2:** Porosity, mean pore size, contact angle and thickness of PSU/PEG substrates.

Substrate	Porosity (%)		*d_p_* (nm)	Contact Angle (°)		Thickness (μm)
	Porous Layer	Substrate		Dry Membrane	Wet Membrane	
PEG-0	80.1 ± 1.0	55.0 ± 1.1	19.0 ± 0.7	94.9 ± 0.7	77.0 ± 3.8	57.8 ± 1.4
PEG4-1	82.9 ± 2.6	55.7 ± 1.5	15.0 ± 1.2	94.0 ± 0.6	72.7 ± 2.0	59.3 ± 0.9
PEG4-2	85.3 ± 1.0	56.8 ± 3.1	14.0 ± 0.8	94.2 ± 1.2	74.1 ± 0.6	62.0 ± 2.2
PEG4-3	85.5 ± 1.3	57.6 ± 0.7	11.8 ± 0.6	93.8 ± 0.8	73.0 ± 1.4	61.4 ± 0.3
PEG4-4	84.6 ± 1.0	56.6 ± 1.4	10.8 ± 0.2	93.3 ± 1.0	73.8 ± 2.1	61.9 ± 1.9
PEG10-4	84.7 ± 1.4	56.8 ± 0.8	9.9 ± 0.5	95.2 ± 1.6	73.1 ± 3.1	62.7 ± 2.2
PEG20-4	84.6 ± 1.9	58.4 ± 2.6	7.8 ± 0.4	95.8 ± 1.0	74.1 ± 2.1	67.0 ± 2.5
PEG4-5	85.7 ± 2.0	57.8 ± 3.2	17.2 ± 0.9	95.3 ± 0.5	75.6 ± 2.9	62.5 ± 2.4
PEG4-6	89.5 ± 1.5	61.5 ± 0.5	23.3 ± 1.1	95.9 ± 0.8	76.1 ± 2.2	61.4 ± 3.4

**Table 3:** XPS surface analysis of PSU/PEG supported TFC-FO membranes.

Membrane	Atomic Composition (%)					O I/O II Ratio	O/N Ratio
	C 1s	N 1s	O 1s	O I	O II		
TFC/PEG-0	74.34	11.78	13.89	84.79	15.21	5.57	1.18
TFC/PEG4-1	74.35	11.51	14.13	87.37	12.63	6.92	1.23
TFC/PEG4-2	73.95	11.31	14.74	83.91	16.09	5.22	1.30
TFC/PEG4-3	74.56	10.90	14.54	83.68	16.32	5.13	1.33
TFC/PEG4-4	74.65	10.64	14.71	81.43	18.57	4.39	1.38
TFC/PEG10-4	74.96	9.78	15.26	72.93	27.07	2.69	1.56
TFC/PEG20-4	74.07	9.59	16.34	62.45	37.55	1.66	1.70
TFC/PEG4-5	73.97	12.22	13.81	85.80	14.20	6.04	1.13
TFC/PEG4-6	74.91	11.21	13.88	85.69	14.31	5.99	1.24

**Table 4:** Roughness of PSU/PEG supported TFC-FO membrane surface.

Membrane	R_q_ (nm)	R_a_ (nm)
TFC/PEG-0	93.6 ± 5.9	75.2 ± 5.0
TFC/PEG4-1	95.0 ± 7.9	75.9 ± 5.4
TFC/PEG4-2	96.8 ± 2.3	77.4 ± 1.6
TFC/PEG4-3	98.1 ± 9.8	78.5 ± 8.3
TFC/PEG4-4	96.2 ± 5.8	76.6 ± 4.5
TFC/PEG10-4	95.1 ± 9.9	75.8 ± 7.3
TFC/PEG20-4	94.2 ± 7.8	74.5 ± 6.1
TFC/PEG4-5	98.0 ± 10.3	78.4 ± 8.5
TFC/PEG4-6	93.2 ± 5.4	73.3 ± 4.2

**Table 5:** Intrinsic properties of PSU/PEG supported TFC-FO membranes.

Membrane	*A* (L m^−2^ h^−1^ bar^−1^)	*B* (L m^−2^ h^−1^)	*A/B* (bar^−1^)	*S* (×10^−6^ m)
TFC/PEG-0	1.26 ± 0.29	0.14 ± 0.02	8.98 ± 1.65	363.7 ± 36.9
TFC/PEG4-1	1.13 ± 0.15	0.15 ± 0.02	8.09 ± 1.65	313.0 ± 9.0
TFC/PEG4-2	1.24 ± 0.15	0.17 ± 0.05	7.75 ± 1.82	300.0 ± 24.3
TFC/PEG4-3	1.28 ± 0.15	0.15 ± 0.04	8.97 ± 2.49	276.0 ± 13.8
TFC/PEG4-4	1.37 ± 0.01	0.17 ± 0.02	8.28 ± 0.80	265.5 ± 7.8
TFC/PEG10-4	1.16 ± 0.07	0.12 ± 0.01	9.43 ± 1.43	253.0 ± 9.0
TFC/PEG20-4	0.61 ± 0.07	0.09 ± 0.02	6.98 ± 2.18	286.7 ± 23.1
TFC/PEG4-5	1.65 ± 0.09	0.17 ± 0.03	9.92 ± 1.35	248.8 ± 18.7
TFC/PEG4-6	1.41 ± 0.23	0.22 ± 0.07	6.68 ± 1.31	242.5 ± 23.7

## Data Availability

Not applicable.

## Supplementary Materials

The following supporting information can be downloaded at: https://www.mdpi.com/article/10.3390/molecules28052318/s1, Table S1: Intrinsic properties of PSU/PEG supported TFC-FO membranes; Figure S1: Water flux of PSU/PEG10 supported TFC-FO membranes; Figure S2: Specific reverse salt flux of PSU/PEG10 supported TFC-FO membranes; Figure S3: Water flux of PSU/PEG20 supported TFC-FO membranes; Figure S4: Specific reverse salt flux of PSU/PEG20 supported TFC-FO membranes; Figure S5: The preparation process images including: (a) before casting, (b) after casting, (c) after phase inversion.
