# Supplementary material for: Effect of Polyethylene Glycol Additive on the Structure and Performance of Fabric-Reinforced Thin Film Composite
Source: Molecules. 2023 Mar 2;28(5):2318. doi: 10.3390/molecules28052318 (PMC10005719; doi:10.3390/molecules28052318)
Supplement: Supplementary file 1 [file molecules-28-02318-s001.zip › molecules-2232101-supplementary.pdf]

Table S1. Intrinsic properties of PSU/PEG supported TFC-FO membranes.

Membrane	A (L m^{-2} h^{-1} bar^{-1})	B (L m^{-2} h^{-1})	A/B (bar^{-1})	S (×10^{-6} m)
TFC/PEG10-1	0.93±0.22	0.14±0.04	6.85±0.95	342.3±37.3
TFC/PEG10-2	1.08±0.21	0.16±0.05	6.95±0.35	296.0±21.7
TFC/PEG10-3	1.12±0.11	0.17±0.02	6.56±1.42	263.3±2.5
TFC/PEG10-4	1.16±0.07	0.12±0.01	9.43±1.43	253.0±9.0
TFC/PEG20-1	0.78±0.04	0.13±0.04	6.66±2.23	368.0±22.1
TFC/PEG20-2	0.71±0.06	0.10±0.00	7.33±0.38	268.0±4.2
TFC/PEG20-3	0.75±0.05	0.09±0.03	8.03±0.57	265.2±7.8
TFC/PEG20-4	0.61±0.07	0.09±0.02	6.98±2.18	286.7±23.1

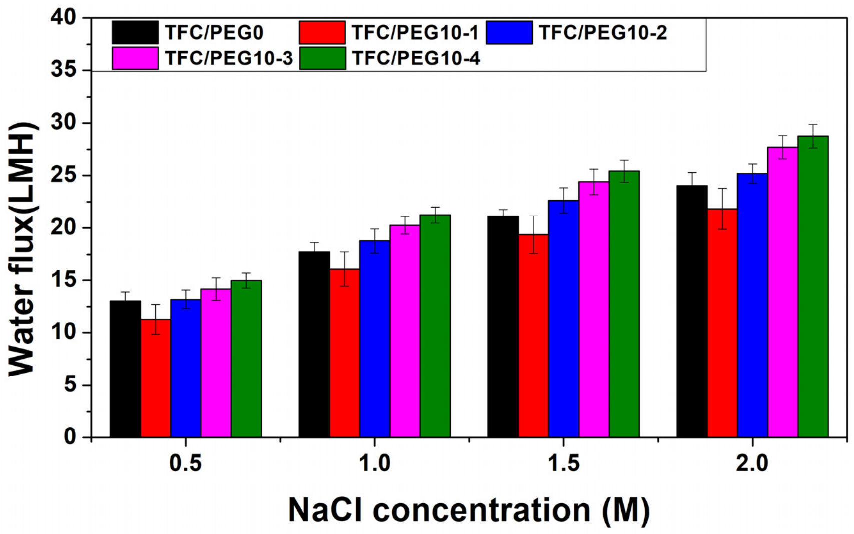

Figure S1. Water flux of PSU/PEG10 supported TFC-FO membranes.

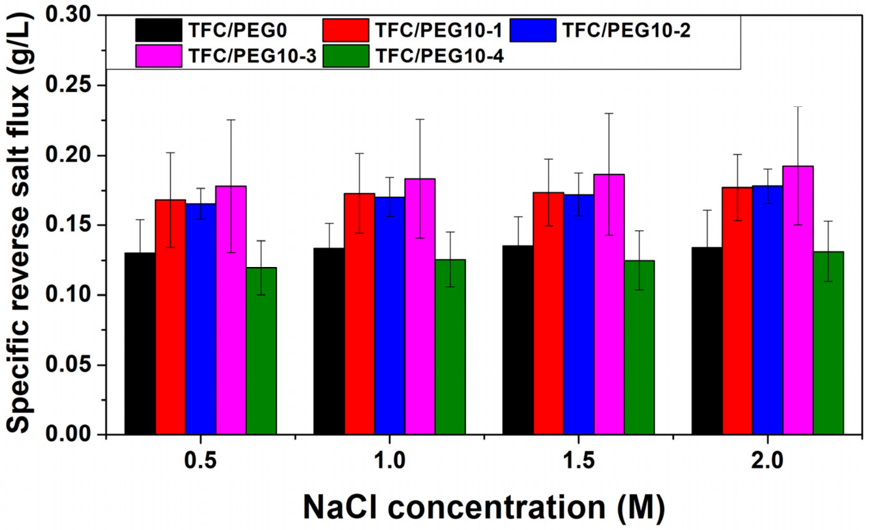

Figure S2. Specific reverse salt flux of PSU/PEG10 supported TFC-FO membranes.

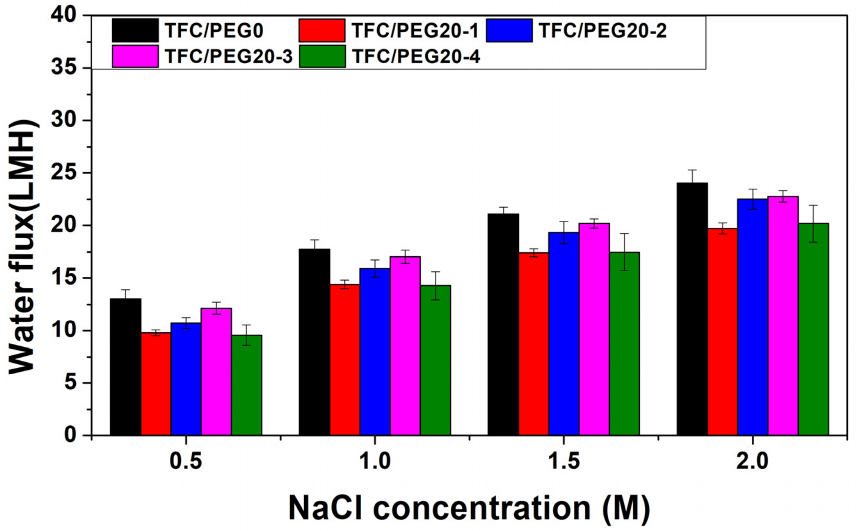

Figure S3. Water flux of PSU/PEG20 supported TFC-FO membranes.

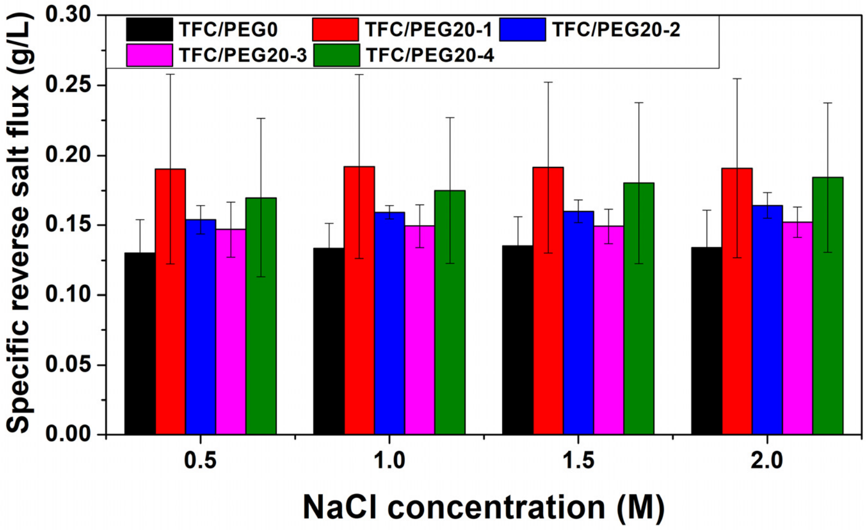

Figure S4. Specific reverse salt flux of PSU/PEG20 supported TFC-FO membranes.

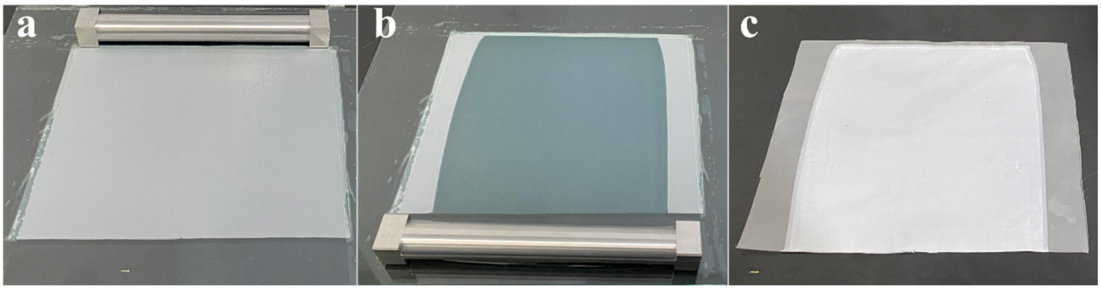

Figure S5. The preparation process images including: (a) before casting, (b) after casting, (c) after phase inversion.
